# Estimation of Foot Trajectory and Stride Length during Level Ground Running Using Foot-Mounted Inertial Measurement Units

**DOI:** 10.3390/s22197129

**Published:** 2022-09-20

**Authors:** Yuta Suzuki, Michael E. Hahn, Yasushi Enomoto

**Affiliations:** 1 Research Center for Urban Health and Sports, Osaka Metropolitan University, 3-3-138 Sugimoto, Sumiyoshi, Osaka 558-8585, Japan; 2 Department of Environmental Physiology for Exercise, Graduate School of Medicine, Osaka Metropolitan University, 3-3-138 Sugimoto, Sumiyoshi, Osaka 558-8585, Japan; 3 Department of Human Physiology, University of Oregon, 181 Esslinger Hall, 1525 University St., Eugene, OR 97403, USA; 4 Faculty of Health and Sport Sciences, University of Tsukuba, 1-1-1 Tennodai, Tsukuba 305-8574, Japan

**Keywords:** running speed, wearable sensors, zero-velocity assumption, gradient descent

## Abstract

Zero-velocity assumption has been used for estimation of foot trajectory and stride length during running from the data of foot-mounted inertial measurement units (IMUs). Although the assumption provides a reasonable initialization for foot trajectory and stride length estimation, the other source of errors related to the IMU’s orientation still remains. The purpose of this study was to develop an improved foot trajectory and stride length estimation method for the level ground running based on the displacement of the foot. Seventy-nine runners performed running trials at 5 different paces and their running motions were captured using a motion capture system. The accelerations and angular velocities of left and right feet were measured with two IMUs mounted on the dorsum of each foot. In this study, foot trajectory and stride length were estimated using zero-velocity assumption with IMU data, and the orientation of IMU was estimated to calculate the mediolateral and vertical distance of the foot between two consecutive midstance events. Calculated foot trajectory and stride length were compared with motion capture data. The results show that the method used in this study can provide accurate estimation of foot trajectory and stride length for level ground running across a range of running speeds.

## 1. Introduction

Running is one of the most popular activities, not only as a sport, but also as a form of recreation all over the world. Although running brings many benefits for runners, running injury risk related to overuse is high [1]. Monitoring runners could be useful for reduction of injury rates, as well as improvement of running performance. This can be achieved through the accumulation of running specific information such as stride frequency and stride length throughout a runner’s daily training program.

Over the past several years, there has been extensive work using inertial measurement units (IMUs) in the field of Biomechanics, especially for gait event detection [2,3,4] and stride length estimation during walking [5] and running [6,7]. Because an IMU measures tri-axial linear accelerations, angular velocities, and magnetic field strength in its own local coordinate system, foot trajectory and stride length in the global coordinate system cannot be estimated directly from the IMU data. Generally, foot trajectory and stride length estimation using IMU data requires two steps; (1) estimate the IMU’s orientation in the global coordinate system from measured angular velocity combined with other sensors (accelerometer or magnetometer), (2) integrate transformed and gravity removed acceleration to estimate the velocity and position of the IMU in the global coordinate system. A main problem with calculating trajectory from IMU data is drift in the acceleration and the angular velocity measurements, limiting position and orientation assessment during long-term measurements. A popular approach to address this drift is a strapdown integration, using a zero-velocity assumption [8,9,10,11], in which the foot is assumed to have a zero-velocity phase (stationary phase) or zero velocity at a specific point during stance regardless of gait speed.

Although the zero-velocity assumption provides a reasonable initialization for integration of acceleration data, noise, bias, and drift in acceleration and angular velocity measurements could remain. The remaining drift will enhance errors in velocity and position estimation during the process of integration. Acceleration-based and velocity-based dedrifting methods combined with the zero-velocity assumption have been used to remove the remaining drift. Several groups have reported that use of dedrifting methods can provide accurate estimation of stride length during walking and running across a range of speeds [12,13,14,15].

The other source of errors for foot trajectory and stride length estimation using IMU data arises from the IMU orientation, because it affects the removal of gravity from acceleration data as well as transformation of acceleration and angular velocity into the global coordinate system. This error is caused not only by the noise and bias of the measured angular velocity but also by the inaccurate estimation of the initial orientation of the IMU [16]. Although the stationary phase plays an important role in the accurate estimation of IMU’s orientation from measured acceleration, this phase becomes rare or nonexistent as running speed increased [14]. These observations indicate that estimation of foot trajectory and stride length would become more difficult as running speed increases. For example, reduction in accuracy of stride length estimation was observed previously for higher running speed [15]. Therefore, it seems that accurate and stable estimation of the IMU orientation is necessary for estimation of foot trajectory and stride length while running over a wide range of speed. For level ground running, it can be assumed that the vertical displacement of the foot between two consecutive support phases should be zero. This assumption could be an effective indicator for the accuracy of IMU orientation estimation.

Therefore, the purpose of this study was to develop an improved foot trajectory and stride length estimation method for the level ground running based on the displacement of the foot, and to investigate the accuracy of the method by comparing outcomes to motion capture data. We also estimated foot trajectory and stride length using the velocity based linear dedrifting method for comparison.

## 2. Materials and Methods

### 2.1. Data Collection and Processing

Seventy-nine runners who runs at least once a week participated in this study approved by the Research Ethics Committee of the Graduate School of Comprehensive Human Science, University of Tsukuba. Written informed consent was obtained before the experiment. All subjects participating in this study were Japanese and their characteristics can be found in Table 1. Subjects having any acute or chronic condition that would limit the ability of the patient to participate in the study were excluded from the study.

**Figure 1:**
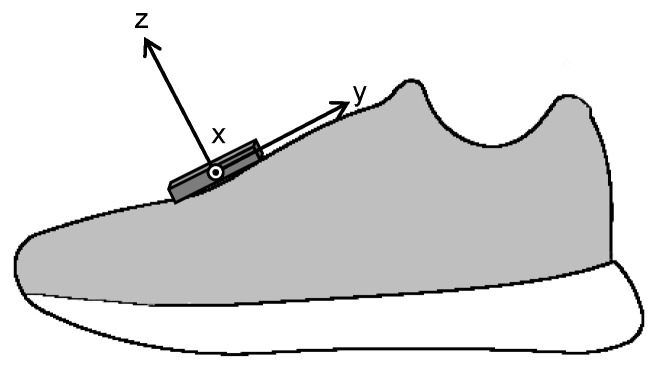
Location of inertial measurement unit and its coordinate system.

The subjects performed running trials at five different paces over a 20 m straight runway and their running motions were collected from at least four of five paces. The running speed of each pace and the number of collected trials are shown in Table 2. Running speed was monitored with photocell sensors set on the runway. Data were captured from approximately 7 m of the last 10 m (10 to 17 m from the start position) using a 16-camera Vicon MX system (Vicon, Oxford, UK, 250 Hz). Thirty-five reflective markers were placed on bony landmarks according to the Plug-in-Gait marker set. In addition, time-synchronized triaxial accelerations (± 16 g) and angular velocities (± 2000 dps) of left and right feet were measured using two IMUs (Casio Computer Co., Ltd., Tokyo, Japan, 200 Hz) fixed to the dorsum of both feet (Figure 1). The coordinates of reflective markers and the accelerations and angular velocities of the foot mounted IMU were low pass filtered using a quintic spline function [17]. The cut-off frequencies were 10 Hz for the marker coordinates and 25 Hz for the IMU data.

**Figure 2:**
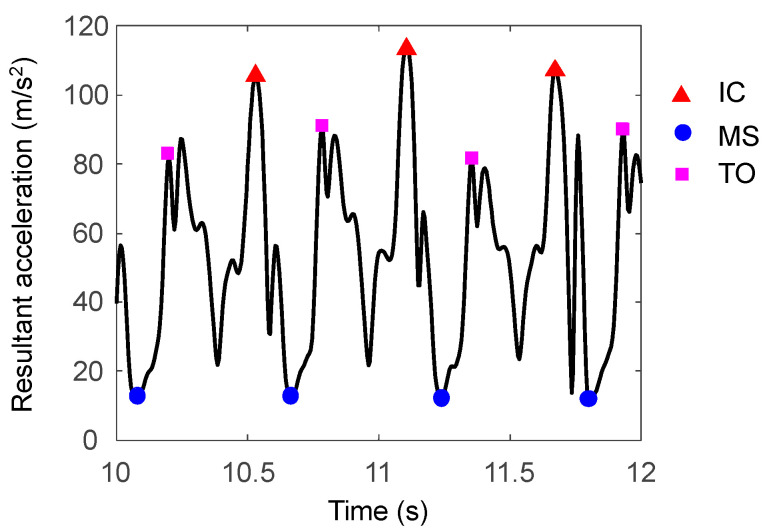
Visualization of detected initial contact (IC), midstance (MS), and toe-off (TO) events using the acceleration data.

**Figure 3:**
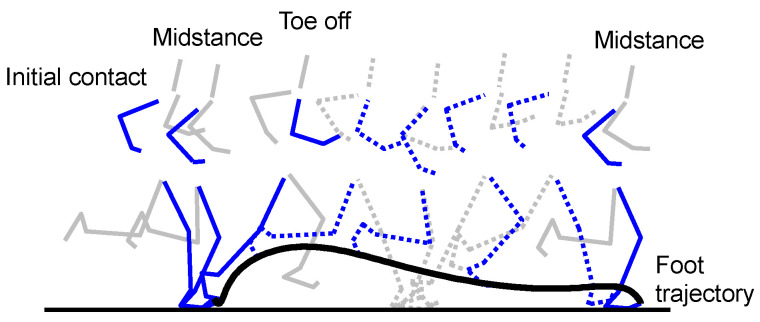
Stick diagrams of running motion and main gait events.

Initial contact (IC) and toe-off (TO) events were detected using acceleration data of the IMU [2,3] (Figure 2). Stick diagrams of running motion and main gait events are shown in Figure 3. Each IC was defined as the instant of the peak resultant acceleration. Each TO was defined as the instant of the 1st peak of resultant acceleration in the time range of 0.1 s to 0.4 s from IC. Midstance (MS) was defined as the instant of minimum resultant acceleration found between IC and TO of the same contact phase. Thus, we regard MS as the instant that only the gravitational acceleration is applied to the IMU.

### 2.2. Foot Trajectory Estimation

#### 2.2.1. Spatial Error Correcting (SEC) Method

**Figure 4:**
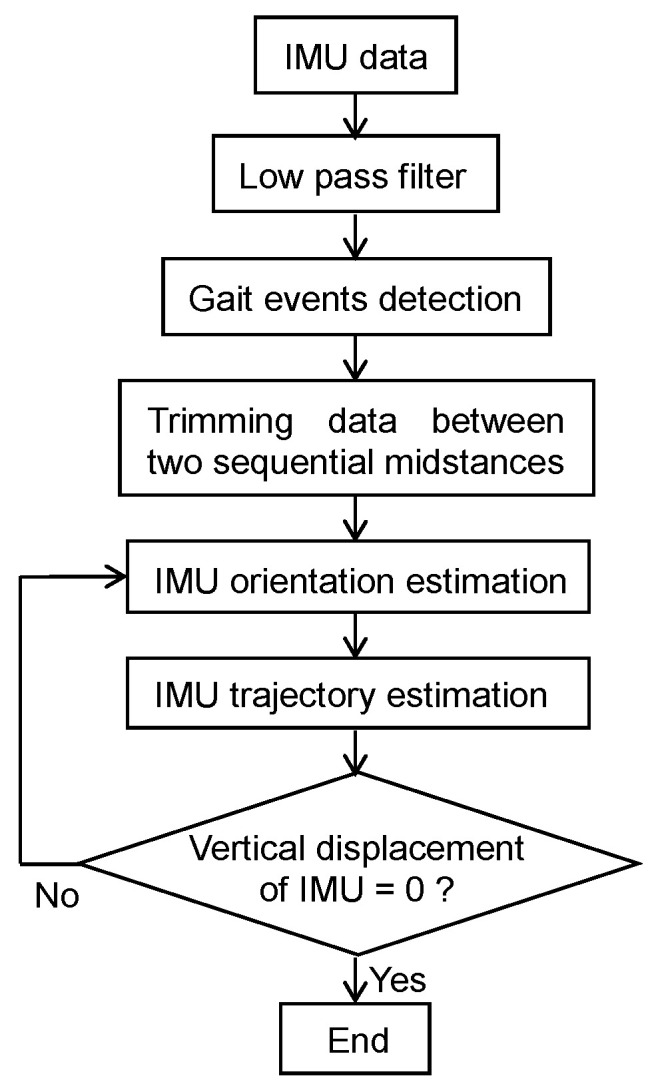
Flow chart of the data postprocessing steps for the estimation of foot trajectory from foot-mounted IMU.

**Figure 5:**
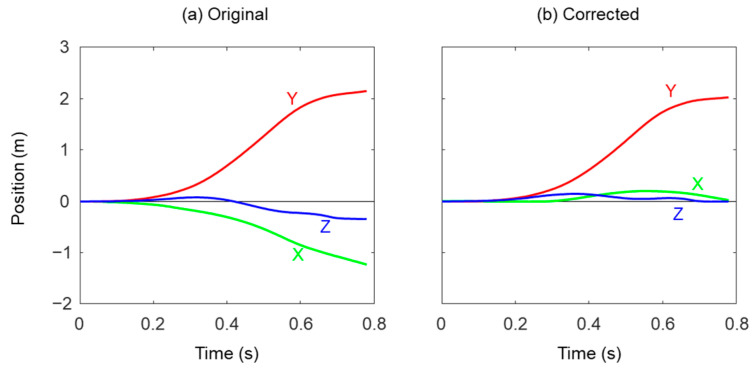
Example of original and corrected foot trajectory obtained from foot mounted IMU of the velocity was used to make both velocities of foot at *MS_i_* and *MS_i_*_+1_ zero. The foot trajectory was then calculated from the dedrifted velocity, and the stride length was calculated from the absolute value of the foot position at *MS_i_*_+1_.

In this study, we estimated foot trajectories between two consecutive and ipsilateral MS instances (*MS_i_* and *MS_i_*_+1_). The process for estimation of foot trajectory from IMU data is shown in Figure 4. Estimation of the foot trajectory starts with first estimating the orientation of the IMU. In this study, we used a quaternion to represent the IMU orientation. Quaternions are defined using four parameters, one defining the angle of rotation and three defining the axis of rotation. The differential of a quaternion is obtained as follows:

(1) $$\dot{q}=-\frac{1}{2}\left[\begin{array}{cccc} 0 & \omega_{z} & -\omega_{y} & \omega_{x}\\ -\omega_{z} & 0 & \omega_{x} & \omega_{y}\\ \omega_{y} & -\omega_{x} & 0 & \omega_{z}\\ -\omega_{x} & -\omega_{y} & -\omega_{z} & 0 \end{array}\right]q$$

where *ω_x_*, *ω_y_*, and *ω_z_* are the angular velocity of the IMU in the global coordinate system. Because an IMU measures the acceleration and angular velocity data in the local coordinate system fixed to the IMU, the data must be transformed into the global coordinate system to calculate foot trajectory. For this transformation, the roll (*ϕ*) and a temporary pitch angles (*θ*) of the IMU at *MS_i_* were calculated as follows:

(2) $$\phi_{MS_{i}}=\tan^{-1}\left(\frac{-a_{x}}{\sqrt{a_{y}{}^{2}+a_{z}{}^{2}}}\right)$$

(3) $$\theta_{MS_{i}}=\tan^{-1}\left(\frac{a_{y}}{a_{z}}\right)$$

where *a_x_*, *a_y_*, and *a_z_* are three components of the measured acceleration at *MS_i_*. Because the yaw angle (*ψ*) cannot be estimated from the acceleration data, it was temporarily set to zero at this stage. After the *MS_i_*, the orientation of the IMU was calculated using the measured angular velocity until the next *MS* (*MS_i_*_+1_). The measured acceleration was transformed into the global coordinate system using the calculated quaternion, and then the gravitational acceleration was removed by subtracting 9.8 m/s^2^ from the vertical component of the transformed acceleration. The foot trajectory was then calculated by integration of the gravity removed acceleration as follows (Figure 5a):

(4) $$v_{g}=v_{0}+\int_{MS_{i}}^{MS_{i+1}}a_{g}dt$$

(5) $$p_{g}=p_{0}+\int_{MS_{i}}^{MS_{i+1}}v_{g}\;dt$$

where *a_g_*, *v_g_*, and *p_g_* are the gravity removed acceleration, velocity, and position of the foot in the global coordinate system, and the initial velocity (*v*_0_) and position (*p*_0_) are set as zero. Assuming that the runners run straight on the level ground in one cycle, the mediolateral and vertical displacements of the foot at *MS*_*i*+1_ should also be zero. Therefore, the yaw angle was calculated from the position of the foot at *MS*_*i*+1_ as follows:

(6) $$\psi_{MS_{i}}=\tan^{-1}\left(\frac{p_{x}}{p_{y}}\right),$$

where *p_x_* and *p_y_* are the mediolateral and anteroposterior positions of the foot at *MS_i_*_+1_. Similarly, the pitch angle was recalculated from the position of the foot at *MS_i+_*_1_. Because the relationship between the pitch angle and vertical position at *MS_i_*_+1_ is nonlinear, a gradient descent method was performed to calculate the pitch angle that makes the vertical position at *MS*_*i*+1_ zero with the initial pitch angle defined as follows:

(7) $${\theta^{\prime }}_{{MS}_{i}}=\tan^{-1}\left(\frac{p_{z}}{\sqrt{p_{x}{}^{2}+p_{y}{}^{2}}}\right)+\theta_{{MS}_{i}}$$

The position of the foot (*p′*) was recalculated from the yaw, roll and pitch angles (Figure 5b). Because the mediolateral (*p′_x_*) and vertical positions (*p′_x_*) at *MS*_*i*+1_ are equal to zero from the SEC calculation method, the stride length was defined as anteroposterior position (*p′_y_*) at *MS*_*i*+1_.

#### 2.2.2. Linear Dedrifting (LD) Based on the Velocity Method

In addition to the SEC method, a LD method was used to calculate foot trajectory for comparison [15]. Similar to the SEC method, the orientation of the IMU at *MS_i_* was estimated from the measured acceleration (Equations (2) and (3)), and gravity was removed from the acceleration in the global coordinate system. A linear function in all three components.

### 2.3. Evaluation

To evaluate the accuracy of LD and SEC methods, the estimated foot trajectory and stride length were compared to those obtained from reflective markers fixed to the left and right toe as reference. Because position data of reflective markers were recorded at 250 Hz, these data were downsampled to 200 Hz.

## 3. Results

**Figure 6:**
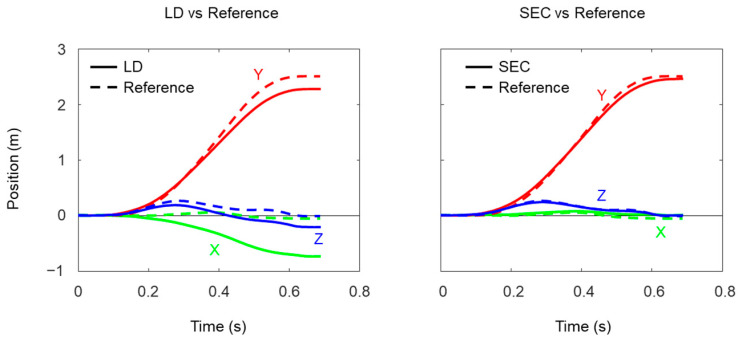
Example of foot trajectories obtained by linear dedrifting (LD) and spatial error correcting (SEC) methods (solid line) with reference (broken line).

A total of 389 trials and 1414 strides (699 strides for the left foot and 715 strides for the right foot) were obtained with both IMUs and VICON in this study. Average running speed was 3.42 ± 0.72 m/s, ranging from 1.86 to 5.89 m/s. Figure 6 shows an example of mediolateral (X), anteroposterior (Y), and vertical trajectories (Z) of the right foot obtained by LD and SEC methods with the reference data. Although correlation coefficients of the Y coordinates were very high both in LD and SEC methods (Table 3), correlation coefficients of the Z coordinates from the SEC method were larger than those from the LD method. In addition, root mean square errors of estimated foot trajectory compared to reference values were smaller in the SEC method than the LD method.

**Figure 7:**
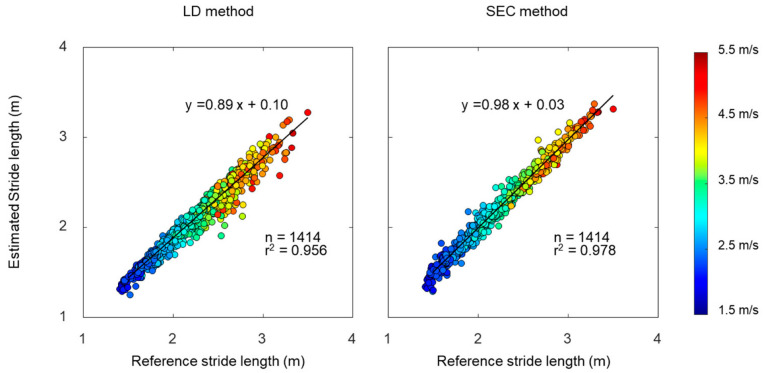
Relationships of stride lengths between estimated by linear dedrifting (LD) and spatial error correcting (SEC) methods and reference.

**Figure 8:**
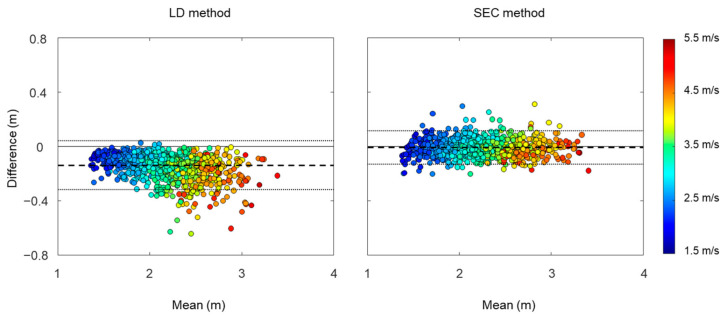
Bland–Altman plot with mean and difference in stride lengths estimated by linear dedrifting (LD) and spatial error correcting (SEC) methods and reference. Limits of agreement are shown as average difference (dashed line) ± 1.96 standard deviation of the difference (dotted line).

The mean stride length was 2.08 ± 0.38 m for the LD method, 2.22 ± 0.41 m for the SEC method, and 2.22 ± 0.42 m for the reference. A linear regression analysis was performed to examine the relationship between estimated stride length and reference stride length (Figure 7). The results of regression analysis show a slightly higher r^2^ in the SEC method compared to the LD method. We used Bland–Altman plots to compare estimated stride length and reference stride length (Figure 8). The offsets were −0.14 m with [−0.32, 0.04] 95% limits of agreement for the LD method, and −0.01 m with [−0.13, 0.11] 95% limits of agreement for the SEC method. 

**Figure 9:**
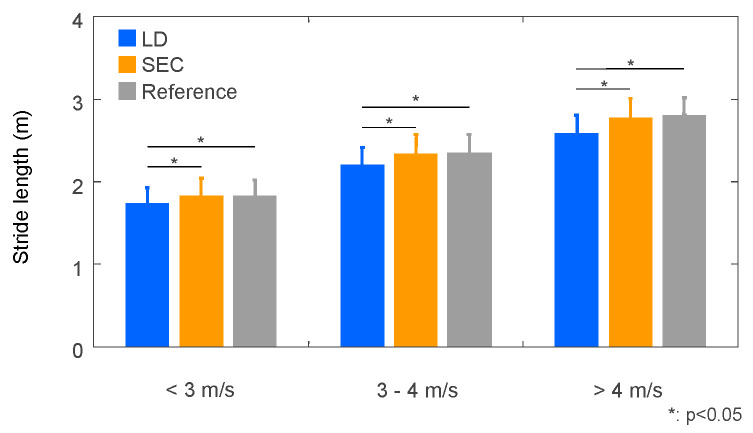
Mean and standard deviation of stride length estimated by linear dedrifting (LD) and spatial error correcting methods (SEC) with measured stride length at different speed ranges.

To investigate the effects of speed range on the stride length estimation, we divided the trials into three groups based on running speed; less than 3 m/s (578 steps), 3 to 4 m/s (571 steps), over 4 m/s (265 steps). The mean stride length of the LD method were smaller than those of the SEC method and the reference in all speed ranges (Figure 9). Errors and absolute errors of stride length compared to the reference were larger for the LD method than for the SEC method in all three speed ranges (Table 4). Intraclass correlation coefficient of the LD method decreased as running speed increased (Table 5), while it did not change with running speed for the SEC method.

## 4. Discussion

The purpose of this study was to develop an improved foot trajectory and stride length estimation method during running on level ground and to investigate the accuracy of the method by comparing its output to motion capture data. The results of the present study showed that the SEC method estimated stride length more accurately compared to the LD method and Zrenner et al. [15]. A primary problem with estimating foot trajectory and stride length from IMU data is drift in the measured accelerations and angular velocities. Because of this, zero-velocity assumption has been used in several previous studies [9,10,11]. Additionally, Glowinski et al. [18] developed an algorithm for estimating drift parameters and orientation of sensors using a motion capture system to reduce the effect of drift. In the present study, the orientation of IMU was estimated with the SEC method and it seems that the effect of IMU drift on stride length estimation was limited in the present study. The results of this study indicate that accurate estimation of IMU orientation may be more important compared to the IMU drift in the estimation of foot trajectory and stride length during running.

For the foot trajectory estimation, small root mean square errors of the SEC method in X and Z directions suggest the assumption that mediolateral and vertical displacements between two consecutive MSs are equal to zero and would be appropriate for the conditions tested in this study. In contrast, larger root mean square errors in X and Y directions were observed in the LD method. This is because the orientation of the IMU at MS was estimated by the acceleration data and the orientation in the horizontal plane cannot be corrected in the LD method. However, the root mean square error in the vertical trajectory was also larger in the LD method than the SEC method. In general, there are two main causes that reduce accuracy of foot trajectory estimation with the zero-velocity assumption. One is modeling error which is caused by nonexistence of zero-velocity instance [11], and the other is measurement error of acceleration and angular velocity [19]. However, small root mean square errors of the SEC method indicate that the zero-velocity assumption still worked in the speed range used in this study. Measurement error causes not only drifting in the velocity data but also inaccurate estimation of the IMU orientation. In principle, the LD method removes drifting from the velocity data, while the SEC method corrects the IMU orientation. These facts suggest the large root mean square error of the LD method could be caused by the inaccurate estimation of IMU orientation.

In general, detecting the direction of gravity from the acceleration data is more difficult as running speed increases, because there is very short or no stationary phase during running at high speed [20]. In addition, orientation error is one of the major sources of drift in calculated velocity and position data [21]. Although orientation of the IMU was calculated from the acceleration minimum during support phase to detect the direction of gravity in this study, no significant differences were observed in the mean stride length between the SEC method and the reference value in any speed range. The errors and intraclass correlation coefficient of the SEC method also showed the stride length was accurately estimated regardless of running speed. These results suggest that the SEC method used in this study could correct the orientation of IMU and provide accurate estimation of stride length across the range of running speed used in this study.

Although correlation coefficients of stride length between the LD method and reference values showed strong relationships, Bland–Altman plots indicated that the LD method tends to underestimate stride length. In addition, errors and intraclass correlation coefficient between the LD method and reference values showed that the underestimation of the LD method became worse as running speed increased. In theory, the dedrifting technique causes reduction in the magnitude of the velocity, because this method subtracts a certain amount of velocity to make the next MS (MS_i+1_) velocity zero. This reduction in the velocity resulted in underestimation of the stride length, especially for higher running speeds.

Despite the LD method using the same algorithm as Zrenner et al. [15], the results of this study showed lower accuracy of the LD method compared to Zrenner et al. [15]; their mean error in stride length estimation was 0.02 m, compared to −0.14 m in the current study. In another study, Zrenner et al. [22] reported that location of IMU affects the accuracy of stride length estimation, and that the dorsum (instep) sensor data showed less accuracy than the cavity sensor which was set in the sole of the shoe under the arch. In their other study, Zrenner et al. [15] mounted the IMU in the sole of the shoe, while the IMU was mounted on the dorsum in this study. In addition, the definition of MS was also different; in Zrenner et al. [15] the MS was defined as the minimum angular velocity during the support phase. The differences in location of IMU and definition of gait events could increase the error in stride length estimation in this study compared to Zrenner et al. [15]. The SEC method is limited to only level ground running due to the assumption that the vertical displacement between two consecutive MS should be zero. Although the SEC method could accurately estimate not only stride length but also foot trajectory during running, we have not yet validated the results in an outside environment, or at faster running speeds (over 5 m/s). Therefore, the findings of the present research may not be directly applicable for these conditions. It would be worthwhile to estimate stride length at faster speeds. Since location and sampling frequency of IMU affects the accuracy of stride length estimation [7,22], future work should evaluate the location and design of IMU for stride length estimation at faster speeds.

In conclusion, this study provides an improved method for foot trajectory and stride length estimation from acceleration and angular velocity data measured by a foot-mounted IMU. Although the basic process of this method is similar to previous studies, the results of the present study demonstrate that the SEC method can provide accurate estimation of foot trajectory and stride length for level ground running across a range of running speeds.

## Figures and Tables

**Table 1:** Characteristics of subjects participating in this study.

	N	Age (Years)	Height (m)	Mass (kg)
Female	22	40.7 ± 12.6	1.59 ± 0.05	49.9 ± 4.7
Male	57	35.1 ± 13.9	1.70 ± 13.9	61.3 ± 6.8

**Table 2:** Running speeds and number of collected trials.

Running Speed (m/s)	Number of Trials
<2.7 m/s	87
2.7~3.3 m/s	102
3.3~3.9 m/s	95
3.9~4.5 m/s	101
>4.5 m/s	26

**Table 3:** Mean and standard deviation of correlation coefficient and root mean square error between foot trajectories estimated with linear dedrifting (LD) and spatial error correcting (SEC) methods and the reference trajectory.

	X	Y	Z
Correlation coefficient			
LD	0.06 ± 0.48	0.99 ± 0.00	0.56 ± 0.27
SEC	0.48 ± 0.44	1.00 ± 0.00	0.97 ± 0.08
Root mean square error (m)			
LD	0.26 ± 0.17	0.17 ± 0.11	0.24 ± 0.20
SEC	0.06 ± 0.04	0.05 ± 0.02	0.03 ± 0.01

**Table 4:** Mean and standard deviation of error and absolute error of estimated stride length by linear dedrifting (LD) and spatial error correcting method compared to the reference.

	LD Method		SEC Method	
	Error	Abs. Error	Error	Abs. Error
<3 m/s	−0.05 ± 0.03 *	0.05 ± 0.03 *	0.00 ± 0.03	0.03 ± 0.02
3–4 m/s	−0.06 ± 0.04 *	0.06 ± 0.04 *	−0.01 ± 0.03	0.02 ± 0.02
>4 m/s	−0.08 ± 0.04 *	0.08 ± 0.04 *	−0.01 ± 0.02	0.02 ± 0.01

*: *p* < 5% compared to SEC.

**Table 5:** Intraclass correlation coefficient (ICC) and confidence interval (CI) of ICC for stride length estimated by linear dedrifting (LD) method and spatial error correcting (SEC) method at different speed ranges.

	LD Method		SEC Method	
	ICC (3, 1)	CI of ICC	ICC (3, 1)	CI of ICC
<3 m/s	0.957	[0.949–0.963]	0.952	[0.944–0.959]
3–4 m/s	0.912	[0.897–0.925]	0.961	[0.954–0.967]
>4 m/s	0.878	[0.847–0.903]	0.968	[0.959–0.975]

## Data Availability

The data presented in this study are available on request from the corresponding author.
